# In-Hospital Outcomes and Temporal Trends of Surgical Versus Intravascular Ultrasound–Guided Endovascular Interventions for Femoropopliteal Disease

**DOI:** 10.1016/j.jscai.2025.102617

**Published:** 2025-03-25

**Authors:** Basel Elsayed, Ahmed Subahi, Hamid Sattar, Amged Abdelaziz, Tahir Mohamed, Omar E. Ali

**Affiliations:** a College of Medicine, QU Health, Qatar University, Doha, Qatar; b Division of Cardiology, Wayne State University/Detroit Medical Center, Detroit, Michigan

**Keywords:** endovascular revascularization, femoropopliteal disease, intravascular ultrasound, peripheral artery disease, surgical revascularization

## Abstract

**Background:**

Peripheral artery disease is a global health concern, with femoropopliteal disease being a common manifestation. Recent advancements in endovascular interventions (EVI), guided by intravascular ultrasound (IVUS), have introduced promising treatment options. This study aims to compare in-hospital outcomes and trends of surgical versus IVUS-guided EVI for femoropopliteal disease.

**Methods:**

The National Inpatient Sample database (2016-2021) was analyzed. Procedures were identified using the International Classification of Diseases, Tenth Revision, Clinical Modification codes, and the Cochran-Armitage test was used to assess temporal trends. Propensity score matching balanced baseline characteristics between the surgical (weighted N = 6700) and IVUS-guided EVI (weighted N = 6700) groups. Multivariable regression analysis adjusting for matched covariates was conducted to compare outcomes.

**Results:**

Multivariable logistic regression revealed that in-hospital mortality was lower in the IVUS-guided EVI group (1.6%) compared to the surgical group (3.5%) (OR, 0.386; 95% CI, 0.216-0.692). IVUS-guided EVI also had significantly fewer periprocedural complications (20.6% vs 24.7%; OR, 0.767; 95% CI, 0.636-0.924), including lower rates of bleeding, shock, infections, wound disruption, and respiratory failure. Multivariable linear regression showed that the length of stay was shorter for the IVUS-guided EVI group (β = –1.7 days; 95% CI, –2.2 to –1.1). No statistically significant differences were observed in inflation-adjusted costs, cardiac complications, major amputation, stroke, or renal failure.

**Conclusions:**

Intravascular ultrasound–guided EVI for femoropopliteal disease are associated with better in-hospital outcomes compared to surgical revascularization, including lower in-hospital mortality, periprocedural complications, and a shorter length of stay. However, future prospective studies are needed to validate these results.

## Introduction

Peripheral artery disease (PAD) is a significant global health issue that profoundly impacts morbidity and mortality rates, particularly affecting the lower limbs and leading to reduced blood flow. In the United States alone, it is estimated that 10 to 12 million individuals are afflicted with PAD, reflecting its extensive reach and clinical significance.^1^ Unlike other atherosclerotic cardiovascular diseases, such as coronary artery disease and stroke, the prevalence and mortality associated with PAD are on the rise,^2^ highlighting the urgent need for increased awareness, early detection, and effective treatment strategies to address this growing burden.

Femoropopliteal disease, which encompasses arterial stenosis of the femoral and popliteal arteries, is a frequent site of PAD. This segment is crucial for supplying blood to the lower limbs, and its involvement often results in debilitating symptoms such as claudication and limb ischemia. The management of PAD typically begins with guideline-based medical therapy and risk factor modifications.^3^ These initial steps aim to alleviate symptoms, improve quality of life, and reduce cardiovascular risk. Despite these efforts, medical therapy alone may not always achieve satisfactory outcomes, necessitating the consideration of more invasive treatment options.

Traditionally, surgical revascularization, such as bypass grafting, has been the mainstay of treatment for severe femoropopliteal disease. Nonetheless, there remains a limited body of data comparing the long-term outcomes of surgical vs endovascular treatments for femoropopliteal disease. A recent meta-analysis of observational studies has shown no significant differences in mortality or amputation rates between the 2 treatment approaches.^4^ However, recent advancements in endovascular interventions (EVI), including percutaneous transluminal angioplasty, stenting, and drug-coated devices, offer less invasive alternatives with potentially shorter recovery times and lower complication rates.^5^^,^^6^ Recent trials, such as BEST-CLI, have compared surgical and endovascular approaches, but there remain limited data specifically comparing these approaches for femoropopliteal PAD.^7^

Intravascular ultrasound (IVUS) has emerged as a valuable tool for optimizing EVI by providing detailed anatomical information, which helps in precise lesion assessment and improving procedural outcomes. IVUS has been shown to reduce the need for repeat revascularization in coronary interventions compared to angiography-guided procedures.^8^ Moreover, recent trials have demonstrated that IVUS significantly improved the outcomes of femoropopliteal EVI, especially when used with drug-coated balloons.^9^^,^^10^ Large observational studies have also demonstrated improved outcomes with IVUS-guided interventions compared to angiography-guided procedures.^11^^,^^12^

Nowadays, consensus statements support the use of IVUS-guided EVI for PAD, highlighting its value in optimizing outcomes.^13^ Despite these findings, direct comparisons between IVUS-guided EVI and surgical revascularization for femoropopliteal disease are still lacking. Therefore, the objective of this study is to compare the in-hospital outcomes and trends of femoropopliteal revascularization procedures, specifically surgical vs IVUS-guided EVI.

## Methods

### Data source

The study examined regional in-hospital outcomes of femoropopliteal disease revascularization using discharge data from the 2016-2021 National Inpatient Sample (NIS) database, part of the Healthcare Cost and Utilization Project (HCUP), sponsored by the Agency for Healthcare Research and Quality.^14^ The NIS is the largest publicly available all-payer inpatient health care database in the United States, covering more than 97% of the US population. The NIS approximates a 20% stratified sample of discharges from US community hospitals, excluding rehabilitation and long-term acute care hospitals. The NIS contains data from around 7 million hospital stays each year when unweighted and estimates around 35 million hospitalizations nationally when weighted. Data within the NIS are derived from billing information submitted by hospitals to statewide data organizations and include comprehensive demographic and clinical characteristics. For this analysis, data from the years 2016 to 2021 were utilized. Institutional review board approval and informed consents were not necessary for this study because the NIS is a publicly accessible data set, and patients are deidentified.

### Study population

This study aimed to identify hospitalizations of adults undergoing revascularization for femoropopliteal disease. The International Classification of Diseases, Tenth Revision, Clinical Modification (ICD-10-CM) system was utilized to analyze diagnoses and procedures, with all fields being queried to categorize the study population. Hospitalizations with PAD diagnosis were identified and those who underwent femoropopliteal revascularization were included. Key exclusion criteria included (1) diagnosis of acute limb ischemia, (2) undergoing hybrid revascularization procedures, and (3) age under 18 years. Those who underwent surgical treatment (bypass grafting or endarterectomy) or IVUS-guided EVI (angioplasty with and without stenting or atherectomy) were considered. The ICD-10-CM codes used in this study are provided in Supplemental Table S1.

### Study outcomes

The primary outcomes of interest were (1) in-hospital mortality and (2) a composite of periprocedural complications. In-hospital mortality was defined as death occurring during the same hospitalization as the revascularization procedure. Periprocedural complications were identified using specific ICD-10-CM codes for intraoperative or postprocedural events, including T81 (complications of procedures, not elsewhere classified), T82 (complications of cardiac and vascular prosthetic devices, implants, and grafts), and I97 (intraoperative and postprocedural complications and disorders of the circulatory system, not elsewhere classified). Temporal trends in the rates of either surgical or IVUS-guided EVI and the corresponding in-hospital mortality were also examined.

Secondary outcomes included length of hospital stay, inflation-adjusted costs, major amputation, bleeding, and specific perioperative complications, including cardiac complications, stroke, renal failure, respiratory failure, shock, infection, and wound disruption. Inflation-adjusted costs were calculated in 2021 US dollars using HCUP cost-to-charge ratios and the US Bureau of Labor Statistics' Consumer Price Index.^15^ Cardiac complications were defined as postprocedural heart failure, cardiac arrest, and myocardial infarction, identified using ICD-10-CM codes under I97.1 (postprocedural cardiac functional disturbances) and I97.7 (intraoperative cardiac functional disturbances). Stroke was defined using codes I97.81 (intraoperative cerebrovascular infarction) and I97.82 (postprocedural cerebrovascular infarction). Postprocedural renal failure and respiratory failure were defined using codes N99.0 and J95.82, respectively. Shock was defined under T81.1, including types such as unspecified (T81.10), cardiogenic (T81.11), and septic (T81.12). Wound disruption was identified using T81.3, which includes external (T81.31), internal (T81.32), and traumatic injury wound repair (T81.33). Postprocedural infections were identified using T81.4, which include superficial surgical site infections (T81.41), deep incisional infections (T81.42), organ/space infections (T81.43), and sepsis (T81.44). Major amputation procedures were included only if coded after the revascularization procedure. Similarly, bleeding events were identified using codes for periprocedural hemorrhage, hematoma, and posthemorrhagic anemia as well as blood transfusion procedures (30233N1), included only if they occurred after the procedure.

### Statistical analyses

All statistical analyses were conducted using Stata 17.0 MP (StataCorp)^16^ and adjusted for the complex survey design of the NIS to produce nationally representative estimates. Descriptive statistics summarized weighted baseline characteristics and outcomes. Continuous variables were reported as medians with interquartile ranges and compared using the design-adjusted Wald’s test. Categorical variables were presented as weighted counts and percentages and compared using the design-adjusted Pearson χ^2^ test. In accordance with HCUP guidelines, counts less than 11 were not reported.

To minimize confounding by baseline characteristics, propensity score matching was employed using the *psmatch2* statistical package. The matching was performed using a 1:1 nearest neighbor method without replacement, and a caliper width of 0.01 times the standard deviation of the logit of the propensity score. The propensity score model included sociodemographic variables (age, sex, race, household income, and primary payer), admission and hospital characteristics (weekend admission, elective admission, region, bed size, and location/teaching status), year of hospitalization, clinical presentation (intermittent claudication [IC], critical limb ischemia [CLI]), and comorbidities identified using ICD-10-CM codes (Supplemental Table S1) and the Elixhauser Comorbidity Software.^17^ Prematching and postmatching balance between groups was assessed using standardized mean differences, with a cutoff of <10% indicating acceptable balance.

For binary outcomes, variables included in the propensity score model were incorporated into multivariable logistic regression models to approximate a doubly robust methodology. This approach was used to evaluate the association between revascularization modality and in-hospital outcomes, with results presented as adjusted odds ratios (OR) and 95% confidence intervals (CI). For continuous outcomes, linear regression analyses were performed, and results were reported as regression coefficients (β) with 95% CI. Statistical significance was defined as a 2-tailed *P* value <.05. Finally, for trend analysis, the Cochran-Armitage test was utilized, with a *P* value <.05 indicating a significant linear trend over the years.

A sensitivity analysis was conducted using 2 approaches. First, the cohort was stratified by clinical presentation into CLI and IC groups, followed by matched analyses within each stratum to compare primary outcomes in surgical vs IVUS-guided EVI. Second, 3 multivariable logistic regression models were compared: (1) surgical vs EVI without IVUS, (2) surgical vs IVUS-guided EVI, and (3) EVI without IVUS vs IVUS-guided EVI, adjusting for covariates including sociodemographic, hospital, clinical, and comorbidity factors.

## Results

### Study population

**Figure 1:**
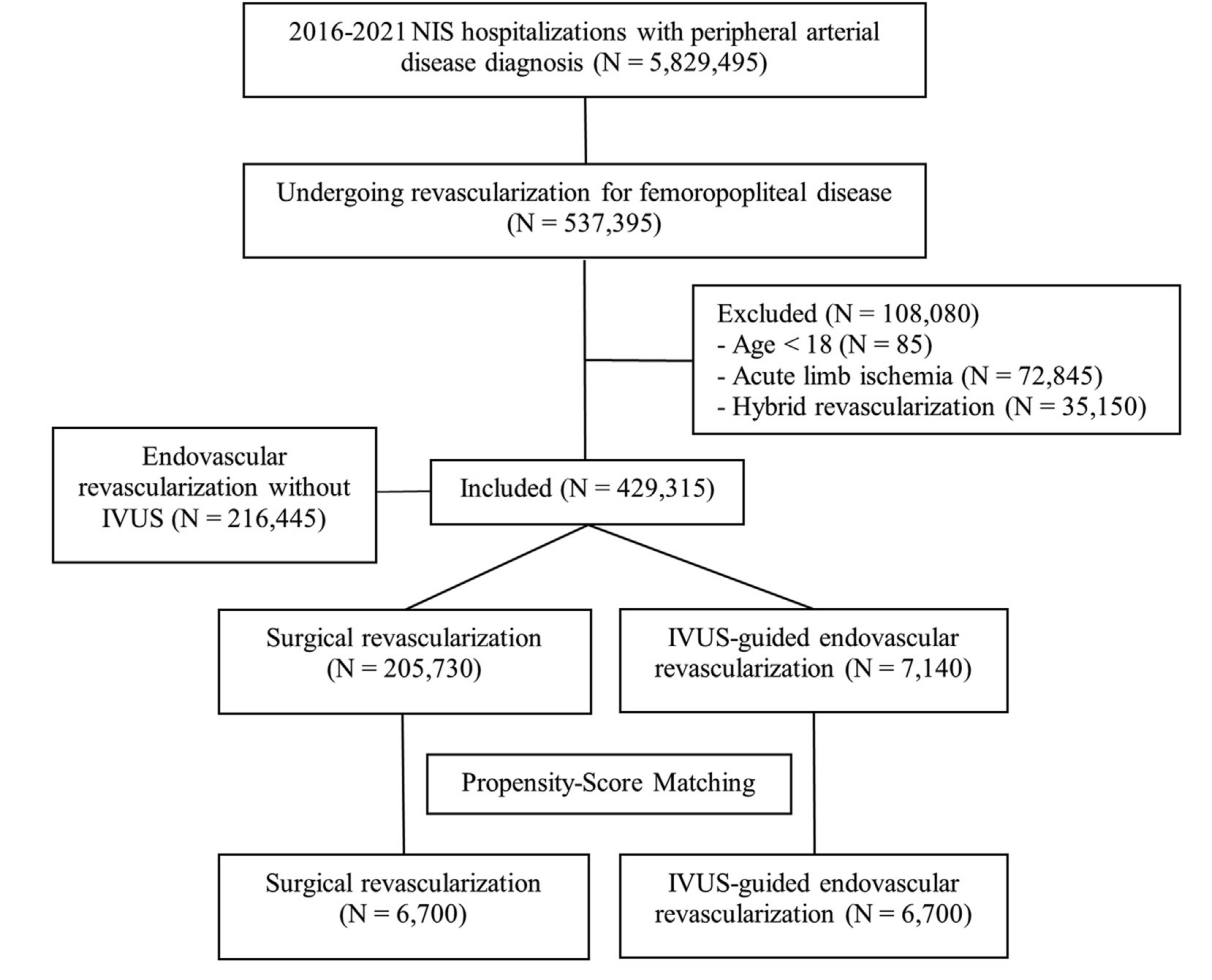
**Flowchart of the study population.** IVUS, intravascular ultrasound; NIS, National Inpatient Sample.

The study included a total of 5,829,495 weighted hospitalizations with a diagnosis of PAD between 2016 and 2021. Among these, 537,395 involved revascularizations for femoropopliteal disease. After applying exclusion criteria, 429,315 hospitalizations were included in the study. Of these, 205,730 underwent surgical revascularization, 216,445 underwent EVI without IVUS, and 7140 underwent IVUS-guided EVI (Figure 1). Among the IVUS-guided EVI group, 6645 involved balloon angioplasties, 4400 with uncoated balloons, and 2245 with drug-coated balloons. Additionally, 1190 used drug-eluting stents, and 2035 used bare-metal stents. Atherectomy and intravascular lithotripsy were performed in 3505 and 105 weighted hospitalizations, respectively.

### Patient characteristics

**Figure 2:**
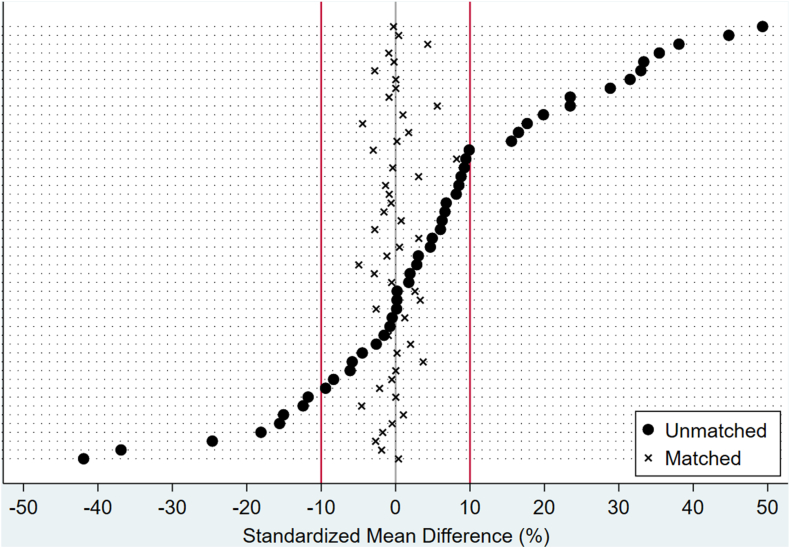
**Balance of baseline characteristicsbefore and after propensity score matching.**

In the unmatched cohort, notable differences emerged between the surgical and IVUS-guided EVI groups (Table 1). The IVUS-guided EVI group had a greater proportion of female patients (42.6% vs 35.0%) and Hispanic individuals (13.9% vs 4.7%). They also were more frequently admitted on weekends (14.5% vs 5.9%). In contrast, the surgical group included more White patients (76.9% vs 60.3%), and a larger proportion of elective admissions (70.4% vs 30.6%). Regionally, IVUS-guided EVI was performed more often in the West (22.7% vs 15.0%). Clinically, the IVUS-guided EVI group presented more frequently with CLI (58.0% vs 42.4%) and had higher rates of several comorbidities, including heart failure (27.2% vs 17.5%), complicated diabetes (56.4% vs 32.6%), complicated hypertension (45.0% vs 28.2%), and severe renal failure (35.7% vs 18.9%). After propensity score matching, the cohort consisted of 6700 weighted hospitalizations in each group, resulting in well-balanced baseline characteristics with all standardized mean differences below 10% (Table 1, Figure 2).

**Table 1:** Baseline characteristics before and after propensity score matching

Variables	Unmatched cohort			Matched cohort		
	Surgical (N = 205,730)	IVUS-guided EVI (N = 7140)	SMD	Surgical (N = 6700)	IVUS-guided EVI (N = 6700)	SMD
Age, y	68 (61-75)	69 (61-77)	9.9%	70 (62-78)	69 (61-77)	–3.0%
Female sex	72,070 (35.0%)	3040 (42.6%)	15.6%	2885 (43.1%)	2890 (43.1%)	0.2%
Race						
White	158,170 (76.9%)	4305 (60.3%)	39.4%	4365 (65.1%)	4200 (62.7%)	2.8%
Black	26,525 (12.9%)	1145 (16.0%)		915 (13.7%)	1110 (16.6%)	
Hispanic	9575 (4.7%)	990 (13.9%)		1005 (15.0%)	950 (14.2%)	
Others	6345 (3.1%)	465 (6.5%)		415 (6.2%)	440 (6.6%)	
Median household income						
0 to 25th percentile	63,540 (30.9%)	2550 (35.7%)	–8.1%	2385 (35.6%)	2450 (36.6%)	–3.5%
26th to 50th percentile	58,985 (28.7%)	1960 (27.5%)		1785 (26.6%)	1845 (27.5%)	
51st to 75th percentile	47,540 (23.1%)	1375 (19.3%)		1375 (20.5%)	1315 (19.6%)	
76th to 100th percentile	32,540 (15.8%)	1130 (15.8%)		1155 (17.2%)	1090 (16.3%)	
Primary expected payer						
Medicare	138,115 (67.1%)	4930 (69.0%)	–4.4%	4530 (67.6%)	4675 (69.8%)	–5.5%
Medicaid	19,445 (9.5%)	775 (10.9%)		700 (10.4%)	710 (10.6%)	
Private insurance	39,550 (19.2%)	1040 (14.6%)		1075 (16.0%)	960 (14.3%)	
Self-pay/others	8335 (4.1%)	380 (5.3%)		395 (5.9%)	355 (5.3%)	
Year						
2016	34,980 (17.0%)	750 (10.5%)	33.4%	670 (10.0%)	690 (10.3%)	–0.6%
2017	33,965 (16.5%)	740 (10.4%)		725 (10.8%)	685 (10.2%)	
2018	32,705 (15.9%)	980 (13.7%)		920 (13.7%)	920 (13.7%)	
2019	35,695 (17.4%)	1375 (19.3%)		1190 (17.8%)	1270 (19.0%)	
2020	32,950 (16.0%)	1375 (19.3%)		1370 (20.4%)	1335 (19.9%)	
2021	35,435 (17.2%)	1920 (26.9%)		1825 (27.2%)	1800 (26.9%)	
Weekend admission	12,055 (5.9%)	1035 (14.5%)	28.9%	925 (13.8%)	925 (13.8%)	0.0%
Elective admission	144,855 (70.4%)	2185 (30.6%)	–87.0%	2095 (31.3%)	2070 (30.9%)	–0.8%
Hospital region						
Northeast	38,640 (18.8%)	1055 (14.8%)	19.9%	1075 (16.0%)	1025 (15.3%)	2.2%
Midwest	50,560 (24.6%)	1505 (21.1%)		1395 (20.8%)	1380 (20.6%)	
South	85,755 (41.7%)	2960 (41.5%)		2755 (41.1%)	2795 (41.7%)	
West	30,775 (15.0%)	1620 (22.7%)		1475 (22.0%)	1500 (22.4%)	
Hospital bed size						
Small	30,905 (15.0%)	1115 (15.6%)	1.1%	870 (13.0%)	1030 (15.4%)	–6.5%
Medium	59,005 (28.7%)	1905 (26.7%)		1780 (26.6%)	1785 (26.6%)	
Large	115,820 (56.3%)	4120 (57.7%)		4050 (60.4%)	3885 (58.0%)	
Hospital location/teaching status						
Rural	10,215 (5.0%)	470 (6.6%)	–11.7%	475 (7.1%)	395 (5.9%)	2.1%
Urban nonteaching	35,005 (17.0%)	1460 (20.4%)		1275 (19.0%)	1355 (20.2%)	
Urban teaching	160,510 (78.0%)	5210 (73.0%)		4950 (73.9%)	4950 (73.9%)	
Clinical presentation						
Intermittent claudication	63,735 (31.0%)	990 (13.9%)	–41.9%	935 (14.0%)	945 (14.1%)	0.4%
Critical limb ischemia	87,265 (42.4%)	4140 (58.0%)	31.5%	2865 (42.8%)	2865 (42.8%)	0.0%
Elixhauser comorbidities						
Heart failure	36,055 (17.5%)	1945 (27.2%)	23.5%	1695 (25.3%)	1850 (27.6%)	5.6%
Deficiency anemias	32,275 (15.7%)	2255 (31.6%)	38.1%	1965 (29.3%)	2085 (31.1%)	4.3%
Valvular disease	15,900 (7.7%)	685 (9.6%)	6.6%	690 (10.3%)	660 (9.9%)	–1.6%
Pulmonary circulation disease	5330 (2.6%)	305 (4.3%)	9.2%	300 (4.5%)	295 (4.4%)	–0.4%
Hypertension, uncomplicated	115,190 (56.0%)	3125 (43.8%)	35.4%	3665 (54.7%)	3695 (55.1%)	–0.9%
Hypertension, complicated	57,915 (28.2%)	3210 (45.0%)	–24.6%	3035 (45.3%)	3005 (44.9%)	–2.7%
Chronic pulmonary disease	69,735 (33.9%)	1910 (26.8%)	–15.6%	1835 (27.4%)	1820 (27.2%)	–0.5%
Diabetes, uncomplicated	13,845 (6.7%)	245 (3.4%)	–15.1%	210 (3.1%)	225 (3.4%)	1.0%
Diabetes, complicated	67,150 (32.6%)	4030 (56.4%)	49.3%	3770 (56.3%)	3760 (56.1%)	–0.3%
Renal failure, moderate	26,810 (13.0%)	1395 (19.5%)	17.7%	1425 (21.3%)	1315 (19.6%)	–4.5%
Renal failure, severe	12,175 (5.9%)	1160 (16.2%)	33.3%	1085 (16.2%)	1080 (16.1%)	–0.2%
Liver disease, moderate	6625 (3.2%)	255 (3.6%)	1.9%	265 (4.0%)	230 (3.4%)	–2.9%
Liver disease, severe	615 (0.3%)	35 (0.5%)	3.1%	35 (0.5%)	30 (0.4%)	–1.2%
Coagulopathy	10,655 (5.2%)	485 (6.8%)	6.8%	455 (6.8%)	445 (6.6%)	–0.6%
Obesity	24,290 (11.8%)	1040 (14.6%)	8.2%	985 (14.7%)	965 (14.4%)	–0.9%
Hypothyroidism	20,215 (9.8%)	840 (11.8%)	6.2%	805 (12.0%)	820 (12.2%)	0.7%
Cerebrovascular disease	18,900 (9.2%)	660 (9.2%)	0.2%	570 (8.5%)	620 (9.3%)	2.6%
Alcohol abuse	10,070 (4.9%)	265 (3.7%)	–5.8%	180 (2.7%)	230 (3.4%)	3.7%
Sum of comorbidities	3 (2, 4)	4 (2, 5)	44.8%	4 (2, 5)	4 (2, 5)	0.4%
Other comorbidities						
Prior angioplasty	11,085 (5.4%)	360 (5.0%)	–1.6%	365 (5.4%)	350 (5.2%)	–1.0%
Prior bypass graft	3545 (1.7%)	140 (2.0%)	1.8%	130 (1.9%)	125 (1.9%)	–0.6%
Coronary artery disease	98,935 (48.1%)	3440 (48.2%)	0.2%	3095 (46.2%)	3205 (47.8%)	3.3%
Smoking	150,935 (73.4%)	4000 (56.0%)	–36.9%	3840 (57.3%)	3780 (56.4%)	–1.9%
Dyslipidemia	134,870 (65.6%)	4655 (65.2%)	–0.8%	4400 (65.7%)	4370 (65.2%)	–0.9%

Values are reported as median (IQR) or weighted counts (%).

EVI, endovascular intervention; IVUS, intravascular ultrasound; N, weighted sample, SMD, standardized mean difference.

### Clinical outcomes

In the propensity-matched cohort, the analysis revealed several significant differences in clinical outcomes between the surgical and IVUS-guided EVI groups (Table 2). The surgical group had a significantly higher in-hospital mortality rate (3.5% vs 1.6%; *P* = .003) and a higher incidence of composite periprocedural complications (24.7% vs 20.6%; *P* = .012) compared to the IVUS-guided EVI group. The surgical group had a longer median length of stay (7 days vs 6 days; *P* < .001), while median inflation-adjusted costs showed no significant difference ($32.2K vs $34.5K; *P* = .860). Moreover, the IVUS-guided EVI had a lower incidence of bleeding (15.0% vs 29.2%; *P* < .001), infections (1.0% vs 1.9%; *P* = .038), shock (0.1% vs 1.0%; *P* = .038), wound disruption (0.5% vs 1.5%; *P* = .005), and respiratory failure (0.1% vs 1.0%; *P* = .003). Other perioperative events, including cardiac complications, major amputation, stroke, and renal failure, did not show statistically significant differences between the groups.

**Table 2:** Comparison of in-hospital outcomes for IVUS-guided endovascular vs surgical interventions in the matched cohort

Outcomes	Surgical (N = 6700)	IVUS-guided EVI (N = 6700)	*P* value
In-hospital mortality	235 (3.5%)	110 (1.6%)	.003^a^
Periprocedural complications	1655 (24.7%)	1380 (20.6%)	.012^a^
Length of stay, d	7 (4, 13)	6 (2, 11)	<.001^a^
Inflation-adjusted costs, in 2021 US dollar	32,244 (19,603-51,723)	34,505 (23,976-51,050)	.860
Cardiac complications	25 (0.4%)	<11	.257
Stroke	20 (0.3%)	<11	.413
Renal failure	20 (0.3%)	<11	.179
Major amputation	140 (2.1%)	100 (1.5%)	.235
Bleeding	1955 (29.2%)	1005 (15.0%)	<.001^a^
Shock	70 (1.0%)	25 (0.4%)	.038^a^
Infection	130 (1.9%)	65 (1.0%)	.038^a^
Wound disruption	110 (1.6%)	35 (0.5%)	.005^a^
Respiratory failure	70 (1.0%)	<11	.003^a^

Values are reported as median (IQR) or weighted counts (%). Cells with a count <11 are not reportable as per the Healthcare Cost and Utilization Project guidelines.

EVI, endovascular intervention; IVUS, intravascular ultrasound; SMD, standardized mean difference.^a^

a Statistically significant (*P* < .05).

### Regression analysis

The propensity-matched multivariable regression analyses revealed several significant results (Table 3, Central Illustration). IVUS-guided EVI was associated with lower odds of in-hospital mortality (aOR, 0.386; 95% CI, 0.216-0.692) and periprocedural complications (aOR, 0.767; 95% CI, 0.636-0.924) compared to surgical revascularization. The IVUS-guided EVI group also had a shorter hospital stay (β, –1.7 days; 95% CI, –2.2 to –1.1). Inflation-adjusted costs showed no significant difference between the groups (adjusted β, $84; 95% CI, –2212 to 2380). Additionally, the IVUS-guided EVI group had lower odds of bleeding (aOR, 0.407; 95% CI, 0.333-0.497), shock (aOR, 0.291; 95% CI, 0.098-0.863), wound disruption (aOR, 0.296; 95% CI, 0.125-0.697), infections (aOR, 0.424; 95% CI, 0.220-0.817), and respiratory failure (aOR, 0.100; 95% CI, 0.020-0.512). No significant statistical differences were observed for cardiac complications, amputation, stroke, or renal failure.

**Table 3:** Regression analysis of in-hospital outcomes for intravascular ultrasound–guided endovascular vs surgical (reference) interventions in the matched cohort

Outcomes	Adjusted^a^ OR (95% CI)/β (95% CI)	*P* value
In-hospital mortality	0.386 (0.216-0.692)	.001^b^
Periprocedural complications	0.767 (0.636-0.924)	.005^b^
Length of stay, d	–1.7 (–2.2 to –1.1)	<.001^b^
Inflation-adjusted costs, in 2021 US dollar	+84 (–2212 to +2380)	.943
Cardiac complications	0.451 (0.059-3.454)	.443
Stroke	0.504 (0.091-2.787)	.432
Renal failure	0.246 (0.028-2.175)	.207
Major amputation	0.728 (0.401-1.322)	.297
Bleeding	0.407 (0.333-0.497)	<.001^b^
Shock	0.291 (0.098-0.863)	.026^b^
Infection	0.424 (0.220-0.817)	.010^b^
Wound disruption	0.296 (0.125-0.697)	.005^b^
Respiratory failure	0.100 (0.020-0.512)	.006^b^

OR, odds ratio; β, regression coefficient.

a Adjusted for sociodemographic variables, admission and hospital characteristics, year of hospitalization, clinical presentation, and comorbidities.

b Statistically significant (*P* < .05).

**Central Illustration:**
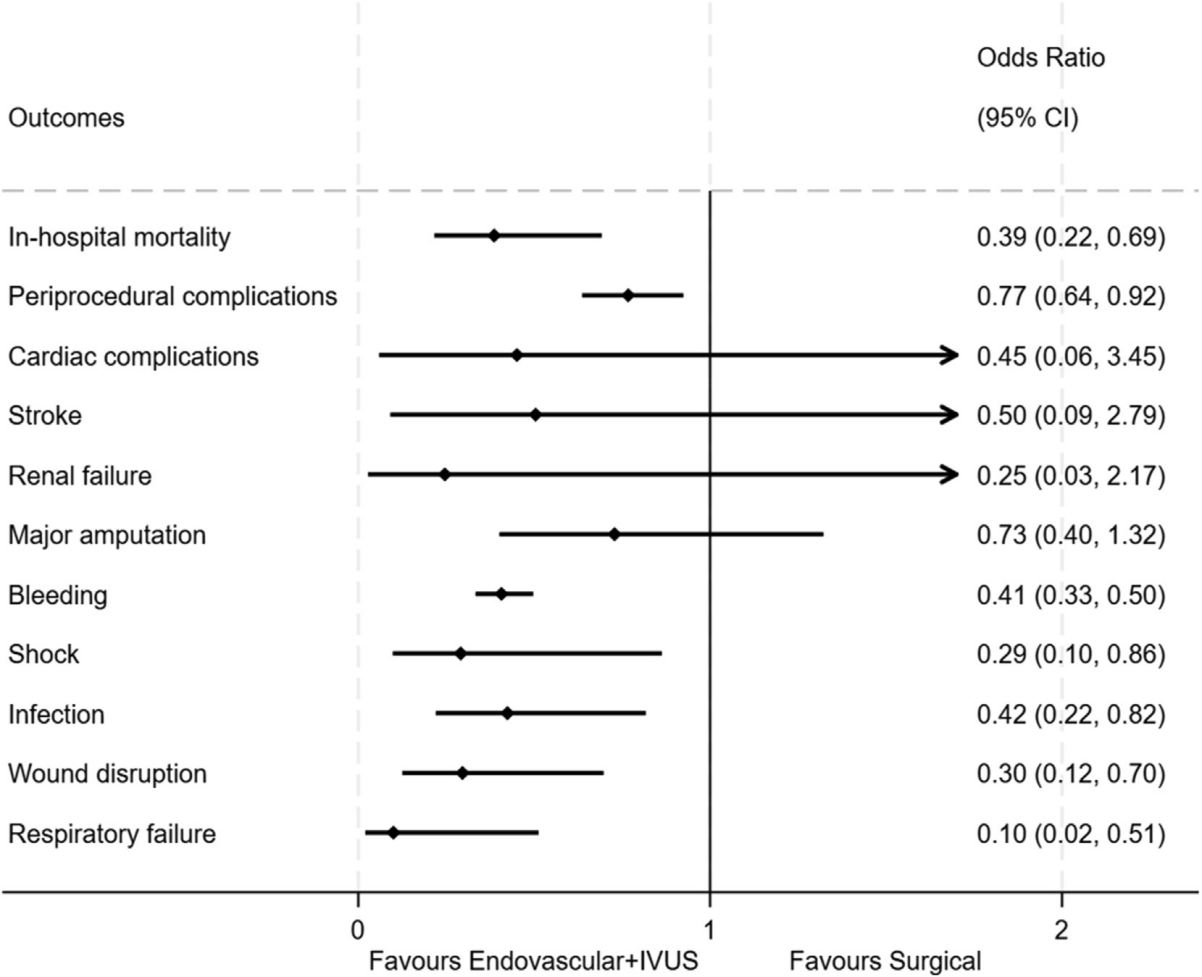
Propensity score–matched multivariable logistic regression analysis of in-hospital outcomes for intravascular ultrasound (IVUS)-guided endovascular vs surgical (reference) interventions.

### Sensitivity analysis

The sensitivity analysis findings are summarized in Table 4, Table 5. Surgical revascularization was associated with higher odds of in-hospital mortality compared to EVI without IVUS (aOR, 0.67; 95% CI, 0.59-0.76) and IVUS-guided EVI (aOR, 0.49; 95% CI, 0.29-0.82), as well as higher odds of periprocedural complications compared to IVUS-guided EVI (aOR, 0.79; 95% CI, 0.68-0.91). No significant differences were found between EVI without IVUS and IVUS-guided interventions in terms of mortality (aOR, 0.74; 95% CI, 0.45-1.21) or complications (OR, 0.95; 95% CI, 0.83-1.09). Second, in the CLI group, IVUS-guided EVI showed lower odds of mortality (OR, 0.69; 95% CI, 0.35-1.34) and complications (OR, 0.84; 95% CI, 0.65-1.10) compared to surgical revascularization. In the IC group, IVUS-guided EVI showed higher odds of mortality (OR, 1.51; 95% CI, 0.24-9.30) and complications (OR, 1.39; 95% CI, 0.87-2.24), though the wide CI for mortality limits interpretability.

**Table 4:** Regression analysis of primary outcomes across surgical, endovascular without IVUS, and IVUS-guided EVI in the unmatched cohort

Outcomes	Surgical (reference) vs EVI without IVUS (N: 205,730 vs 216,445)^a^	Surgical (reference) vs IVUS-guided EVI (N: 205,730 vs 6700)^a^	EVI without IVUS (Reference) vs IVUS-guided EVI (N: 216,445 vs 6700)^a^
In-hospital mortality	aOR, 0.67; 95% CI, 0.59-0.76; *P* < .001^b^	aOR, 0.49; 95% CI, 0.29-0.82; *P* = .007^b^	aOR, 0.74; 95% CI, 0.45-1.21; *P* = .229
Periprocedural complications	aOR, 1.05; 95% CI, 1.01-1.10; *P* = .020^b^	aOR, 0.79; 95% CI, 0.68-0.91; *P* = .002^b^	aOR, 0.95; 95% CI, 0.83-1.09; *P* = .477

aOR, adjusted odds ratio; EVI, endovascular intervention; IVUS, intravascular ultrasound; N, weighted sample.

a Adjusted for sociodemographic variables, admission and hospital characteristics, year of hospitalization, clinical presentation, and comorbidities.

b Statistically significant (*P* < .05).

**Table 5:** Regression analysis of primary outcomes for IVUS-guided endovascular vs surgical (reference) interventions stratified by clinical presentation and matched within each stratum

Outcomes	Critical limb ischemia Surgical (reference) vs IVUS-guided EVI (N: 850 vs 850)^a^	Intermittent claudication Surgical (reference) vs IVUS-guided EVI (N: 3760 vs 3760)^a^
In-hospital mortality	OR, 0.69; 95% CI, 0.35-1.34; *P* = .274; N = 115 vs 80	OR, 1.51; 95% CI, 0.24-9.30; *P* = .655; N = <11 vs 15
Periprocedural complications	OR, 0.84; 95% CI, 0.65-1.10; *P* = .202; N = 780 vs 680	OR, 1.39; 95% CI, 0.87-2.24; *P* = .169; N = 200 vs 255

EVI, endovascular intervention; IVUS, intravascular ultrasound; N, weighted sample; OR, odds ratio.

a IVUS-guided EVI and surgical intervention groups were propensity-matched using sociodemographic variables, admission and hospital characteristics, year of hospitalization, and comorbidities.

### Trend analysis

**Figure 3:**
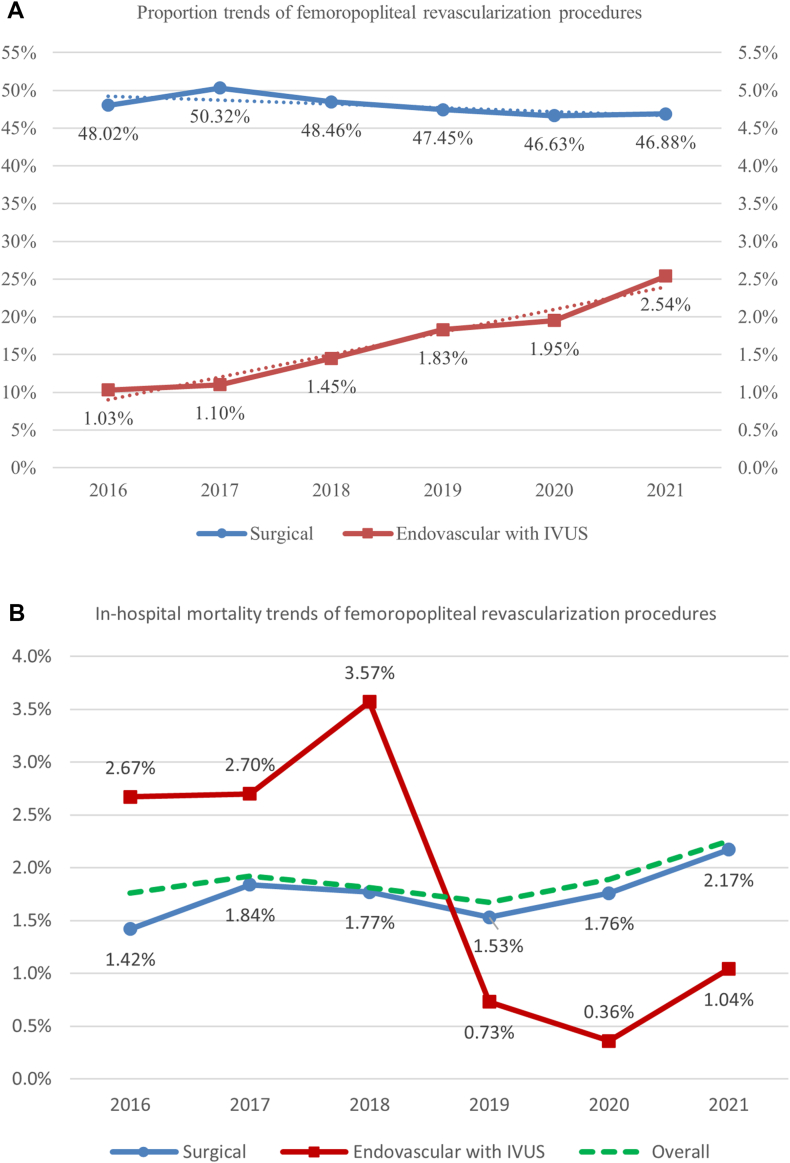
(**A**) Temporal trends in the utilization of femoropopliteal revascularizations. (**B**) Temporal trends of in-hospital mortality for femoropopliteal revascularization. IVUS, intravascular ultrasound.

The proportion of femoropopliteal IVUS-guided EVI group has shown a significant upward trend over the study period (Figure 3A), starting at 1.03% in 2016, and, reaching 2.54% in 2021 (*P*-trend < 0.001). In contrast, the proportion of surgical revascularization procedures initially rose from 48.02% in 2016 to 50.32% in 2017. However, from 2018 onwards, there was a steady decline, with the proportion decreasing to 46.88% in 2021 (*P*-trend < 0.001). Similarly, in-hospital mortality trends for both surgical and IVUS-guided EVI revealed significant variations (Figure 3B). The mortality rate for surgical procedures showed many fluctuations; however, the overall linear trend was upward, starting at 1.42% in 2016 and peaking at 2.17% in 2021 (*P*-trend = 0.037). Conversely, the in-hospital mortality rate for IVUS-guided EVI showed a different pattern. It started at 2.67% in 2016 and peaked at 3.57% in 2018. It then significantly decreased to 0.73% in 2019 and remained low at 0.36% in 2020 and 1.04% in 2021 (*P*-trend = 0.001).

## Discussion

In this study, we analyzed 537,395 hospitalizations in the United States between 2016 and 2021 involving patients diagnosed with PAD and undergoing femoral-popliteal revascularization. We observed a significant increase in the use of IVUS-guided EVI, which correlated with a decreasing trend in in-hospital mortality. Furthermore, IVUS-guided EVI was associated with a reduced risk of periprocedural complications, including respiratory failure, shock, infection, and wound disruption, when compared to surgical revascularization.

The increasing use of IVUS-guided EVI aligns with findings from other studies, which reported similar trends using other databases.^18^^,^^19^ Divakaran et al^11^ observed a moderate increase in IVUS use during deep venous stent placement among Medicare beneficiaries from 2017 to 2019. Moreover, EVI has become widely adopted as the first line of revascularization for femoral-popliteal PAD, and improvements in operator skills and technology have allowed for the management of increasingly complex lesions. This shift is reflected in expert consensuses and updated guidelines, including the Inter-Society Consensus for the Management of Peripheral Vascular Arterial Disease,^20^ the 2017 European Society of Cardiology guidelines recommending EVI for femoral-popliteal lesions <25 cm^3^, and the 2024 ACC/AHA guidelines^1^ endorsing endovascular therapy as a class I recommendation for femoral-popliteal PAD and claudication.^13^^,^^21^

Nonetheless, the proportion of femoropopliteal procedures performed surgically remains substantial, reflecting either provider preference or the complexity of certain lesions. By including a non-IVUS endovascular arm in our sensitivity analysis, our study confirms that although endovascular therapy itself confers an in-hospital mortality advantage over surgery, the use of IVUS may further refine procedural strategies and contribute to improved outcomes. Although EVI without IVUS also showed advantages, the addition of IVUS further enhanced outcomes, particularly in reducing perioperative complications. Moreover, stratified analysis by clinical presentation offered further insights. In the CLI group, IVUS-guided EVI showed a trend toward better outcomes compared to surgical revascularization, likely reflecting the greater procedural precision required in this complex population. In the IC group, IVUS-guided EVI was associated with higher odds of mortality and complications compared to surgery, though this was not statistically significant and the wide CI for in-hospital mortality limits its interpretability. These findings align with the XLPAD registry, which reported that CLI patients frequently present with multilevel or below-the-knee disease and may derive particular benefit from IVUS due to its ability to optimize treatment strategies.^18^ However, these trends warrant further investigation to elucidate the underlying mechanisms and confirm whether they persist in larger, prospective data sets.

Endovascular intervention offers several advantages over surgical revascularization, including a minimally invasive approach, lower hemodynamic challenges, and moderate sedation, all of which contribute to lower periprocedural complications and shorter hospital stays. Considering these factors, along with improvements in operator skills and technology, the observed lower mortality associated with IVUS-guided EVI can be explained. However, the year-to-year variability in mortality may also be influenced by the relatively small sample size of this subgroup, resulting in larger statistical fluctuations. Additionally, evolving operator experience, changes in institutional adoption rates of IVUS, and variations in patient selection (eg, more complex or critically ill individuals receiving IVUS in certain years) could further contribute to the observed fluctuations. Future studies with larger data sets and prospective designs will be crucial to validate these findings.

Our study uniquely compares real-world outcomes of IVUS-guided EVI, highlighting its impact on improving procedural success compared to surgical revascularization. IVUS provides cross-sectional imaging of the vessel wall, addressing the limitations of angiography, which is constrained by its single-plane projection of 3-dimensional vessel anatomy. By offering better visualization of lesion morphology, severity, and length, IVUS improves procedural planning and reduces radiation exposure and contrast use, thereby mitigating the risk of contrast-induced nephropathy.^22^ IVUS surpasses angiography in evaluating lesion eccentricity, calcification, and hemodynamic significance. It also provides a superior assessment of vessel diameter, leading to more accurate sizing for balloons, stents, and other equipment.^23^^,^^24^ Furthermore, IVUS can be used to identify situations in which further vessel preparation (eg, atherectomy or lithotripsy) may be needed.^25^ Postintervention, IVUS can reveal inadequate stent expansion or malposition, as well as procedural complications, including arterial dissections, for which angiography has limited sensitivity.^26^^,^^27^

Available literature suggests that these advantages translate into improvements in periprocedural as well as long-term outcomes. In the recently published IVUS-DCB trial, 237 patients undergoing angioplasty for symptomatic femoral-popliteal disease were randomized to IVUS plus angiography or angiography alone. After 1 year, primary patency was significantly higher in the IVUS group (83.8% vs 70.1%; HR, 0.46; 95% CI, 0.25-0.85; *P* = .01),^10^ with a particular benefit in complex Trans-Atlantic Inter-Society Consensus–defined C and D lesions (*P* = .002). IVUS patients were less likely to need target-lesion revascularization and more likely to experience sustained clinical improvement. The use of IVUS allowed for larger predilation balloon diameters, higher dilation pressures, and more frequent postdilation. Similarly, in 2022, Allan et al^9^ demonstrated in a randomized trial that IVUS-guided angioplasty resulted in higher freedom from binary restenosis at 12 months (72.4% vs 55.4%; *P* = .008). The study also reported that IVUS led to changes in treatment strategies in 79% of cases, further improving outcomes, particularly in drug-coated balloon-treated patients (restenosis 9.1% vs 37.5%; *P* = .001). A retrospective analysis of US beneficiaries undergoing peripheral arterial interventions from 2016 to 2019 also supported these findings, showing better long-term outcomes with IVUS.^7^

The NIS provides a large, validated, and nationally representative database, but our study has several limitations. The retrospective design and reliance on ICD-10-CM codes introduce the potential for unmeasured confounding and limit access to critical clinical details such as procedure indications, lesion complexity, localization, and success rates. Although the stratified sensitivity analysis separating CLI and IC cohorts addressed disease-state heterogeneity, residual confounding remains a possibility. Key data points, such as medication use, preprocedural amputation planning, and comorbidity severity, were unavailable, potentially influencing treatment decisions. Additionally, the absence of ICD-10 codes for subsequent or repeat revascularizations during the index hospitalization restricted the analysis of procedural patterns. Although the inclusion of a non-IVUS endovascular comparison group in the sensitivity analysis provided insight into the role of IVUS, the lack of detailed procedural data limits the ability to fully quantify its specific impact. Prospective studies with more comprehensive data sets are needed to evaluate the sustained clinical benefits and outcomes of IVUS-guided interventions.

The differences observed in our real-world data warrant further analyses in prospective studies to replicate these findings and determine whether greater utilization of IVUS in EVI practice can result in improved procedural success. Moreover, the algorithm for employing IVUS-derived media-to-media diameter for coronary stent sizing is well established, but it is not well-defined for peripheral EVI. Future studies should be directed at defining how to size vessels to optimize luminal gain with balloons and for stent implantation. Finally, although IVUS can outperform angiography in detecting procedure-related dissections, there is no universal consensus on when to treat IVUS-detected dissections.^28^

## Conclusion

In this study, we found that IVUS-guided EVI for femoropopliteal disease are associated with significantly better in-hospital outcomes compared to traditional surgical revascularization. Specifically, the use of IVUS was linked to lower mortality rates, fewer periprocedural complications, and shorter hospital stays. Sensitivity analyses further highlighted the consistent advantages of IVUS-guided EVI compared to EVI without IVUS, while also revealing variations in outcomes when stratified by clinical presentation. These findings emphasize the potential of IVUS-guided EVI as a safer and more effective alternative to surgery, particularly in patients with CLI or complex vascular anatomy. However, further research is needed to confirm these results and to develop standardized guidelines for the use of IVUS in peripheral arterial interventions.

## Footnotes

To access the supplementary material accompanying this article, visit the online version of the *Journal of the Society for Cardiovascular Angiography & Interventions* at 10.1016/j.jscai.2025.102617.
