# Supplementary material for: In-Hospital Outcomes and Temporal Trends of Surgical Versus Intravascular Ultrasound–Guided Endovascular Interventions for Femoropopliteal Disease
Source: J Soc Cardiovasc Angiogr Interv. 2025 Mar 25;4(6):102617. doi: 10.1016/j.jscai.2025.102617 (PMC12230467; doi:10.1016/j.jscai.2025.102617)
Supplement: Supplemental Table S1 [file mmc1.pdf]

Supplemental Table S1. International Classification of Diseases, Tenth Edition, Clinical Modification (ICD-10-CM) codes to identify diagnoses, procedures, comorbidities and outcomes.

Variable	ICD-10-CM code
Diagnoses	
Peripheral artery disease	I70.2*, I73.9*
Acute limb ischemia	I74.3*, I75.02*
Critical limb ischemia	I70.22*, I70.23*, I70.24*, I70.25*, I70.26*
Intermittent claudication	I70.211, I70.212, I70.213
Procedures	
Surgical revascularization	041K09H, 041K0AH, 041K0JH, 041K0KH, 041K0ZH, 041K49H, 041K4AH, 041K4JH, 041K4KH, 041K4ZH, 041K09L, 041K0AL, 041K0JL, 041K0KL, 041K0ZL, 041K49L, 041K4AL, 041K4JL, 041K4KL, 041K4ZL, 041L09J, 041L0AJ, 041L0JJ, 041L0LJ, 041L0ZJ, 041L49J, 041L4AJ, 041L4JJ, 041L4LJ, 041L4ZJ, 041L09L, 041L0AL, 041L0JL, 041L0LL, 041L0ZL, 041L49L, 041L4AL, 041L4JL, 041L4LL, 041L4ZL, 04CK0ZZ, 04CL0ZZ, 04CM0ZZ, 04CN0ZZ
Endovascular revascularization	047K*, 047L*, 047M*, 047N*, X27H*, X27J*, X27K*, X27L*, X27M*, X27N*, 04CK3ZZ, 04CL3ZZ, 04CM3ZZ, 04CN3ZZ
Intravascular ultrasound	B44FZZ3, B44GZZ3, B44HZZ3, B44LZZ3
Comorbidities**	
Previous bypass graft	I70.3*, I70.4*, I70.5*, I70.6*, I70.7*
Previous angioplasty	Z98.62*, Z95.820*
Smoking	Z72.0*, Z87.891*, F17.2*
Dyslipidemia	E78*
Coronary artery disease	I25*
Outcomes	
Periprocedural complications	I97*, T81*, T82*
Cardiac complications	I97.121, I97.711, I97.191, I97.791, I97.111, I97.131
Stroke	I97.81*, I97.82*

**Variable**	**ICD-10-CM code**
Renal failure	N99.0*
Major amputation	0Y62*, 0Y63*, 0Y64*, 0Y6M0Z0, 0Y6N0Z0, 0Y67*, 0Y68*, 0Y6C*, 0Y6D*, 0Y6F*, 0Y6G*, 0Y6H*, 0Y6J* (only if coded after revascularization)
Bleeding	I97.418, I97.42, I97.618, I97.638, D62 + 30233N1* (only if coded after revascularization)
Shock	T81.1*
Infection	T81.4*
Wound disruption	T81.3*
Respiratory failure	J95.82*

** All other comorbidities were identified using the Elixhauser Comorbidity Software (v2022.1).
